# A Low-Cost Two-Dimensional Scalable Active Receive Phased Array with 8 Simultaneously Reconfigurable Beams

**DOI:** 10.3390/mi17030348

**Published:** 2026-03-12

**Authors:** Haifu Zhang, Li-Xin Guo, Shubo Dun, Xiaoming Li, Wei Mei, Xiaolong Xu, Dinuo Bu

**Affiliations:** 1 School of Physics, Xidian University, Xi’an 710071, China; zhflpp@163.com; 2 54th Research Institute of CETC, Shijiazhuang 050081, China; 3932100132@139.com (S.D.); adamcti@163.com (X.L.); xuxiaolongphd@163.com (X.X.); dinuo_bu@foxmail.com (D.B.); 3 Laboratory of Electromagnetic Space Cognition and Intelligent Control, Beijing 100089, China; mwr0326@163.com; 4 School of Physics, University of Electronic Science and Technology of China, Chengdu 611731, China

**Keywords:** phased array, dual circular polarization, multi-beam, analog beamforming network

## Abstract

This paper presents a compact multi-beam dual-circularly polarized phased array receiving system operating in the 10.7–12.7 GHz frequency band is designed and implemented, which can generate eight reconfigurable receiving beams with independently configurable polarization modes and scanning directions for each beam. To improve the aperture utilization efficiency of the array and reduce the array size, the proposed phased array architecture adopts a “full-aperture multiplexing” beamforming method, where all beams share the same array aperture. For cost-effective phased array architecture with two-dimensional scalability, the array is divided into several identical receiving subarrays, with the control and power supply modules arranged beneath the array aperture. In addition, a heterogeneous integration scheme is introduced to realize high-density integration of various receiving functional chips, which reduces the overall array footprint by approximately 30% while maintaining the basic performance of the system gain-to-noise-temperature ratio (G/T). Meanwhile, different dielectric substrates are adopted to implement multi-level combining networks, optimizing the trade-off between overall efficiency and cost. To verify the feasibility of the proposed architecture, a prototype with a 16 × 16 array configuration is developed and tested. The measured results show that the array gain reduction is no more than 4 dB at a maximum scanning angle of 60°, and the G/T value of all beams in the boresight direction is not less than 0.9 dB/K at 11.7 GHz. The experimental results validate the effectiveness of the proposed multi-beam dual-circularly polarized phased array architecture in terms of engineering implementation and system performance.

## 1. Introduction

With the rapid advancement of technology and the increasing demands of both military and civilian applications, phased array antennas have attracted significant attention due to their advantages in beam agility, adaptive beamforming, and low radar cross section (RCS) [1]. Conventional phased array antennas typically generate only a single independent beam at a time, which cannot satisfy the requirements of certain application scenarios. For instance, in multi-target tracking [2] and direction finding [3], multi-beam phased arrays provide higher data throughput and can significantly enhance tracking accuracy and direction-finding precision. In satellite communications [4,5,6], multi-beam phased arrays enable satellites to simultaneously receive signals from multiple terminals located in different directions, thereby greatly improving spectrum utilization.

To meet these requirements, various types of multi-beam antennas have been proposed in the literature [7,8,9,10,11,12,13,14,15,16,17,18,19,20,21,22,23,24,25,26,27,28]. According to their operating principles, multi-beam antennas can generally be classified into three categories: reflector-based multi-beam antennas [7,8,9,10,11], lens-based multi-beam antennas [12,13,14,15,16,17], and multi-beam phased arrays [18,19,20,21,22,23,24,25,26,27,28]. Multi-beam antennas based on reflectors or lenses typically generate multiple beams by placing several feed sources in the focal region, with each feed corresponding to a specific beam. However, the number of feeds is inherently limited by the available physical space, making it difficult for reflector- or lens-based multi-beam antennas to support a large number of beams. In addition, their relatively low aperture efficiency, limited beam scanning capability, and bulky structural profiles further restrict their applicability in large-scale systems.

The beamforming network (BFN) is one of the key components of a multi-beam phased array. Beamforming networks can be broadly categorized into digital BFNs, analog BFNs, and hybrid analog–digital BFNs. In addition to these conventional electronic implementations, emerging beamforming approaches based on optical true-time-delay networks, microwave photonic beamformers, and integrated photonic beamforming processors have also been reported in recent years [29,30]. Digital BFNs provide the highest degree of freedom and the most powerful beamforming capabilities. However, for large-scale phased arrays, the prohibitively high cost and substantial computational burden associated with digital BFNs hinder their widespread deployment. Compared with fully digital architectures, hybrid analog–digital BFNs offer a better balance between cost and complexity, but they are typically limited to one-dimensional electronic scanning or restricted scanning ranges. Analog BFNs can be further divided into fixed analog BFNs and arbitrary (reconfigurable) analog BFNs. Typical fixed analog BFNs include the Butler matrix [31], Nolen matrix [32], and Blass matrix [33]. In multi-beam arrays employing fixed analog BFNs, each beam generally points toward a predetermined direction, and the design complexity increases significantly as the array size and number of beams grow. In contrast, multi-beam phased arrays based on arbitrary analog BFNs allocate a dedicated set of electronically controllable transmit/receive (T/R) components to each beam, enabling fully independent two-dimensional beam scanning. The main challenge associated with this architecture lies in integrating multiple sets of T/R modules within a limited physical volume while maintaining low implementation cost.

This work proposes a general low-cost implementation framework for an eight-beam dual-circularly polarized (DCP) phased array based on a reconfigurable analog BFN. A DCP multi-beam active receiving phased array operating in the 10.7–12.7 GHz frequency band is designed and implemented, capable of generating eight reconfigurable receiving beams with independently configurable polarization modes and scanning directions. The array adopts a full-aperture beam-multiplexing scheme, identical block-partitioned receiving subarrays, and distributed control and power supply modules, thereby enabling a phased array architecture with improved aperture efficiency, reduced physical size, two-dimensional scalability, and a high cost–performance ratio. Heterogeneous integration of the receiving modules reduces the array footprint by nearly 30% without significant degradation in the gain-to-noise-temperature ratio (G/T). Meanwhile, hierarchical combining networks implemented on different dielectric substrates optimize the trade-off between efficiency and cost. Measured results of the fabricated 16 × 16 prototype demonstrate that the gain degradation is less than 4 dB when the array scans to 60°, and the boresight G/T values of all beams are no lower than 0.9 dB/K at 11.7 GHz, which verifies the feasibility and effectiveness of the proposed architecture.

The remainder of this paper is organized as follows. Section 2 presents the operating principle and implementation approach. Section 3 describes the prototype architecture and configuration. Section 4 reports the measurement setup and experimental results. Finally, Section 5 concludes the work.

## 2. Operating Principle and Implementation Approach

**Figure 1:**
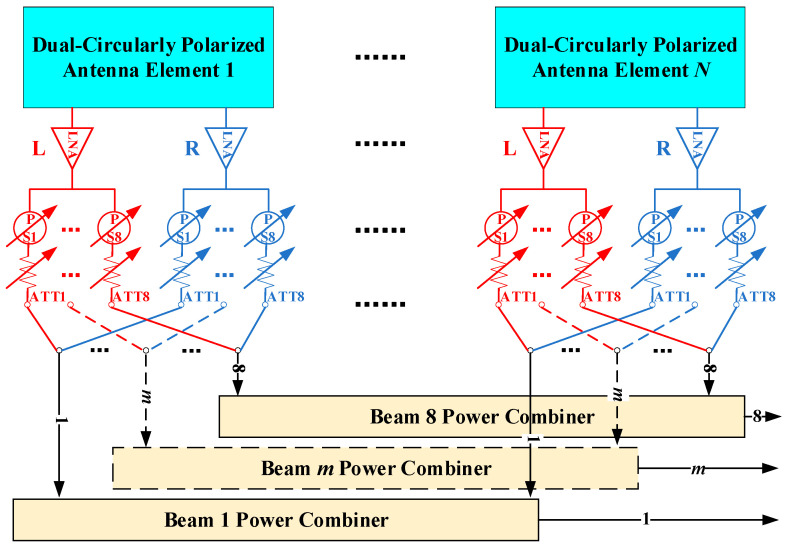
Schematic diagram of generating eight independent and switchable dual-circularly polarized beams using dual-circularly polarized antennas.

**Figure 2:**
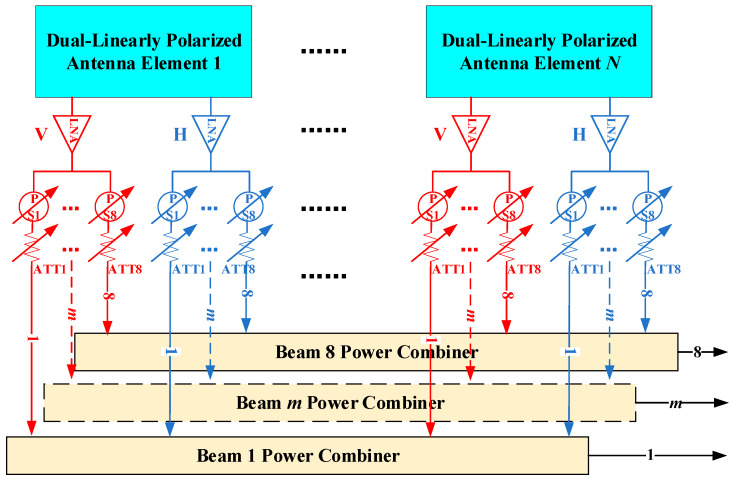
Schematic diagram of generating eight independent and switchable dual-circularly polarized beams using dual-linearly polarized antennas.

Figure 1 and Figure 2 respectively show the schematic diagrams of the operating principle for realizing eight mutually independent and switchable DCP receiving beams based on DCP antennas and dual-linearly polarized (DLP) antennas. In both structures, each polarization port of every antenna array element is connected to a low noise amplifier (LNA), so as to reduce the noise figure (NF) of the radio frequency (RF) front-end under the condition of the same array aperture size, thereby improving the overall G/T value of the system.

To form eight mutually independent receiving beams, each polarization signal is split into eight channels after amplification by the LNA. To ensure that all eight beams possess fully independent electronic scanning and beamforming capabilities, an independently digitally controlled phase shifter and attenuator are configured at each output port of the power divider in both architectures. While the two architectures are quite similar in their overall design philosophy, there remain several important differences in their specific implementation schemes.

In the first architecture, as illustrated in Figure 1, eight switches are required for each array element to select the desired polarization mode for each beam, thereby enabling independent polarization control of the eight beams. After polarization selection, the signals from all array elements are combined for each beam through its corresponding synthesis network. In contrast, the second architecture, shown in Figure 2, does not employ polarization selection switches. Instead, the two orthogonal linear polarization channels of each element simultaneously participate in the signal synthesis process of every beam. Circular polarization is achieved by introducing a phase difference of ±90° between the two linear polarization channels of the same array element. It should be noted that when the phased array system is required to support a large instantaneous bandwidth, additional true time delay (TTD) units can be incorporated into the synthesis network to suppress beam squint and dispersion effects that may occur under large-angle scanning conditions.

Figure 1 presents the structural schematic of an eight independent and switchable DCP beam implementation based on DCP antennas, while Figure 2 shows the corresponding implementation based on DLP antennas. The two beamforming methods exhibit distinct characteristics. Compared with the DLP-based solution, the DCP-based approach requires only half the synthesis network complexity. In the DLP architecture, each circularly polarized beam is synthesized from two orthogonal linear-polarized channels, resulting in duplicated amplitude–phase control paths. In contrast, the DCP-based method directly generates LHCP/RHCP at the element level, thereby avoiding redundant beamforming chains. This structural difference reduces the number of beamforming and combining ICs, RF interconnections, and associated bias/control circuits, leading to lower power consumption, simplified control network design, improved layout compactness, and enhanced thermal performance. The reduced channel count also alleviates integration density and assembly complexity in heterogeneous SIP implementation, contributing to improved manufacturability and overall cost effectiveness. In addition, when all beams operate under the same polarization mode, half of the LNAs can be deactivated, further improving energy efficiency.

However, the DCP-based approach has two limitations. First, the polarization states are restricted to LHCP/RHCP, whereas the DLP-based architecture can theoretically realize arbitrary polarization states including VHP/HP. Second, the electric bridge required for circular polarization formation introduces additional insertion loss (approximately 1 dB), which may slightly reduce passive element efficiency. For Ku-band multi-beam satellite communication systems, this polarization restriction has limited practical impact, as LHCP or RHCP operation is typically required. Moreover, the DLP-based architecture may introduce additional insertion loss and RF chain noise due to its duplicated beamforming paths. Under identical aperture conditions, the two methods therefore exhibit comparable overall G/T performance. Considering performance, integration complexity, and system-level cost collectively, the DCP-based beamforming architecture is selected for the proposed array design.

**Figure 3:**
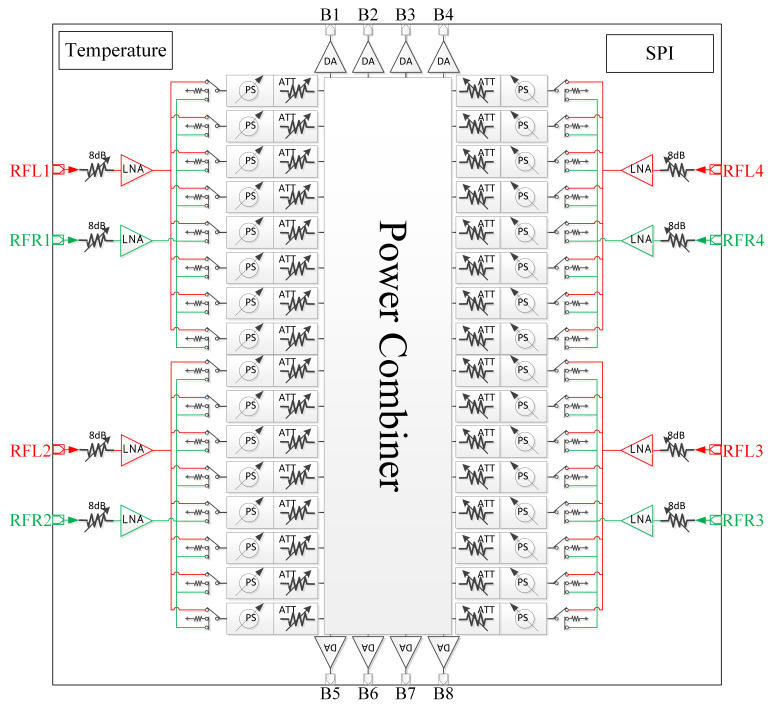
Schematic diagram of the multifunctional chip.

**Figure 4:**
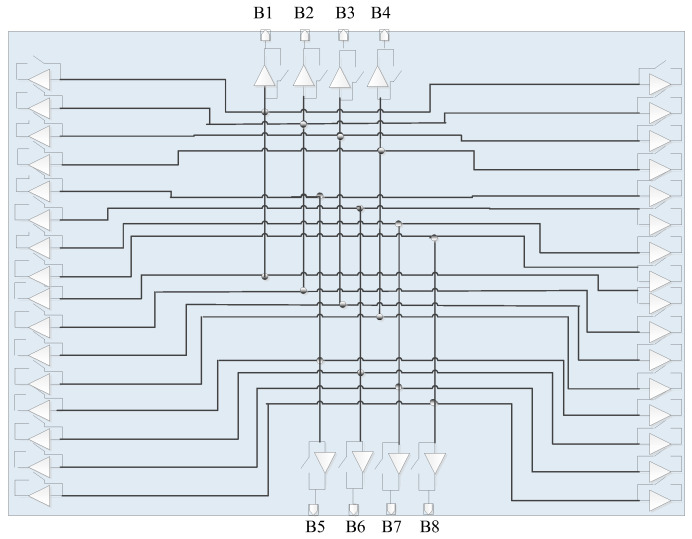
Schematic diagram of the power dividing/combining chip.

Figure 3 shows the schematic diagram of the multifunctional chip. The main challenge in implementing the eight-beam array illustrated in Figure 1 is how to integrate eight mutually independent RF links and their corresponding control and power supply lines within a limited physical space. To address this problem, two types of key chips are designed and developed in this work. Figure 3 presents an eight-beam four-channel multi-functional chip, which is mainly used to implement polarization selection and beamforming for four antenna array elements. Figure 4 shows an eight-beam four-channel power dividing/combining chip, which integrates eight 4-in-1 combiners and one 1-to-4 power divider in a single chip. By controlling the switch states, the chip can operate in either combining mode or dividing mode, and the reserved dividing function can lay a foundation for the subsequent development of the eight-beam array.

Both types of the aforementioned chips are implemented based on silicon-based processes, which offer significant cost advantages under large-scale production conditions. However, the NF of silicon-based chips is generally higher than that of compound semiconductor devices. To further reduce the antenna volume and system cost, compound semiconductor chips can be introduced at the front stage of the multi-functional chips to lower the overall noise figure of the RF links.

On the premise of having the above chip foundation, it is also necessary to select an appropriate substrate material to achieve interconnection between chips. In view of the advantages of high-temperature co-fired ceramic (HTCC) in thermal conductivity and interconnection capability, HTCC is selected as the main interconnection substrate in this work. Nevertheless, with the increase in array scale, the manufacturing yield of HTCC modules may decrease. Therefore, in the subsequent high-level synthesis stage, lower-cost printed circuit board (PCB) substrates can be adopted to strike a balance between manufacturing cost and system performance.

## 3. Prototype Architecture and Configuration

### 3.1. Overall Architecture

**Figure 5:**
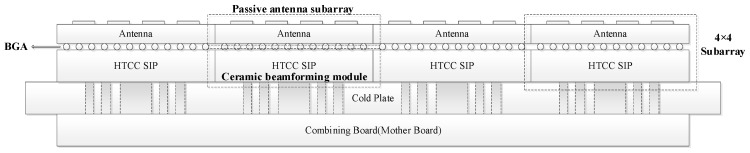
Cross-sectional architecture of the proposed eight-beam dual-circularly polarized receiving phased array prototype.

**Figure 6:**
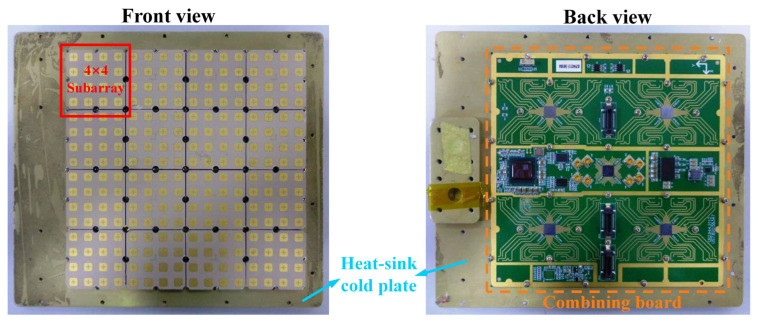
Photograph of the fabricated eight-beam dual-circularly polarized receiving phased array prototype.

Figure 5 presents the overall cross-sectional architecture of the proposed eight-beam DCP receiving phased array prototype, and Figure 6 shows the photograph of the fabricated proof-of-concept prototype. The developed array mainly consists of 16 receiving subarrays with a size of 4 × 4, a heat dissipation cold plate and a combining board. To facilitate subsequent array scale expansion and engineering application, the system adopts a modular and scalable architecture design.

As shown in the cross-sectional structure, the receiving subarrays, heat dissipation cold plate, and combining board are vertically integrated in a stacked configuration. The receiving subarrays are located at the top layer and are responsible for Ku-band signal reception and primary beamforming. The heat dissipation cold plate is arranged beneath the receiving subarrays, providing mechanical support and efficient thermal management for the active devices. The combining board is positioned at the bottom layer to realize higher-level signal combining among multiple subarrays, as well as the decoding and distribution of beam control codes and system power management. This vertically integrated layered architecture not only ensures excellent radio-frequency performance and thermal management capability, but also effectively reduces the overall profile height of the array, thereby improving the compactness and engineering feasibility of the system.

### 3.2. Receiving Subarray Design

**Figure 7:**
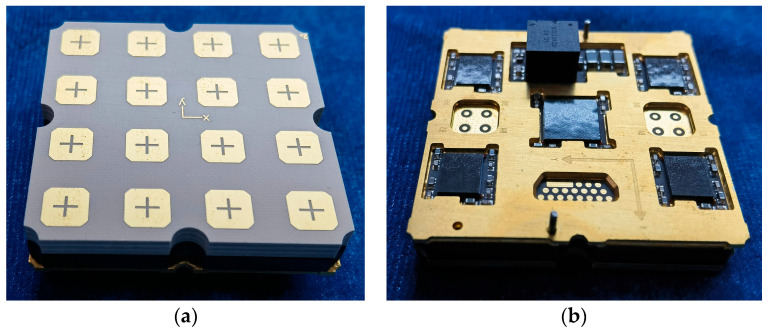
Photograph of the fabricated receiving subarray. (**a**) Front view of the passive antenna subarray; (**b**) Back view of the ceramic beamforming module.

**Figure 8:**
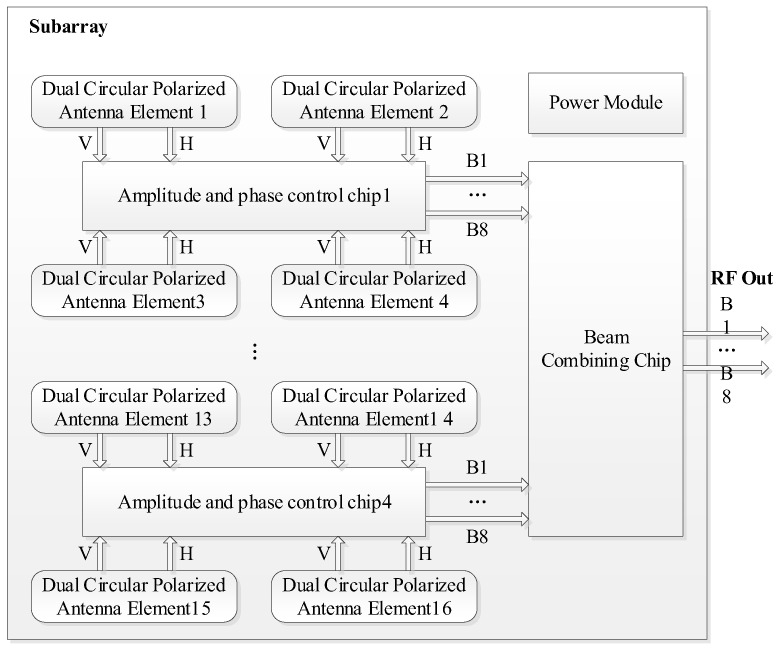
System-level block diagram of the receiving subarray.

Each receiving subarray consists of an antenna subarray and a ceramic beamforming module, which are interconnected via ball grid array (BGA) solder balls to achieve high-density integration. Figure 7 presents a photograph of the fabricated receiving subarray, where Figure 7a shows the front view of the antenna subarray and Figure 7b shows the back view of the ceramic beamforming module. The corresponding system-level functional block diagram of the receiving subarray is illustrated in Figure 8.

**Figure 9:**
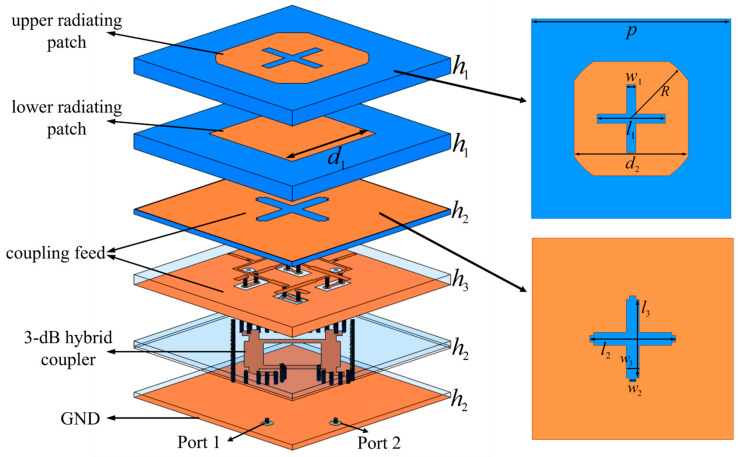
Configuration of the dual-layer slot-coupled microstrip patch antenna element used in the passive antenna subarray. *p* = 10.5 mm, *h*_1_ = 0.762 mm, *h*_2_ = 0.254 mm, *h*_3_ = 0.508 mm, *d*_1_ = 5.6 mm, *d*_2_ = 6 mm, *l*_1_ = 3.6 mm, *l*_2_ = 4.5 mm, *l*_3_ = 4.18 mm, *w*_1_ = 0.5 mm, *w*_2_ = 0.35 mm, *w*_3_ = 0.68 mm.

The antenna structure is illustrated in Figure 9 and operates over the 10.7–12.7 GHz frequency band. The element adopts a planar microstrip patch configuration with a two-layer aperture-coupled feeding scheme. A two-layer dielectric substrate structure is employed. Both dielectric substrates are fabricated from GNC3002 material, featuring a relative permittivity of 2.94 and a loss tangent of 0.002. The two substrate layers are bonded using a prepreg material with a relative permittivity of 2.8. A 3 dB directional coupler is employed in the feeding network to realize broadband dual-circularly polarized radiation. The coupler contains two input ports and two output ports. When Port 1 is excited and Port 2 is terminated with a matched load, the two output ports exhibit equal amplitudes with a +90° phase difference, thereby generating LHCP. Conversely, when Port 2 is excited and Port 1 is terminated with a matched load, the two output ports maintain equal amplitudes with a −90° phase difference, enabling RHCP. The two output ports of the coupler are further cascaded with two aperture-coupled feeding branches. The signals are coupled to the upper radiating patch through microstrip lines and slot coupling to realize antenna radiation. Since the antenna ground plane must be precisely aligned with the radio-frequency interfaces of the rear ceramic module, an impedance transformation transition structure is introduced between the antenna RF ports and the chip interfaces. This transition structure ensures geometric alignment while maintaining good impedance matching performance.

**Figure 10:**
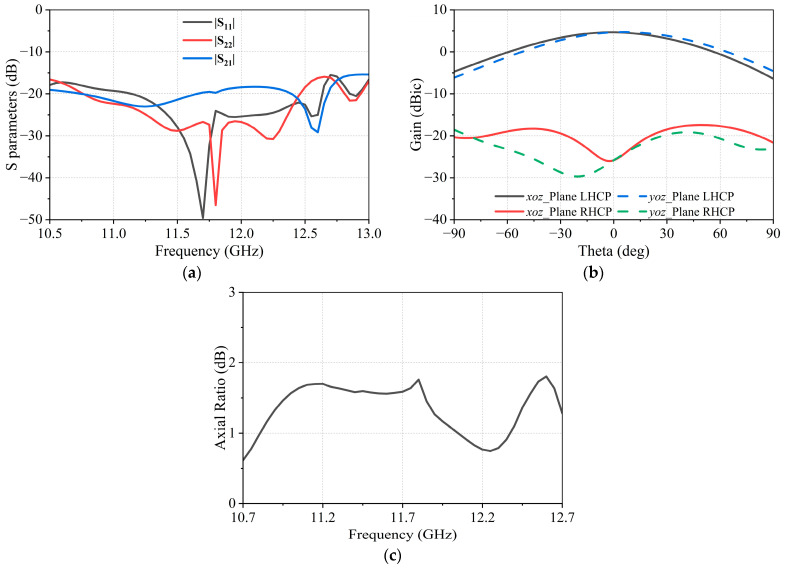
Simulated performance of the antenna element. (**a**) S parameters; (**b**) Radiation pattern at 11.7 GHz; (**c**) Axial Ratio.

The simulation results of the antenna element are presented in Figure 10, which show that the antenna exhibits favorable impedance matching characteristics and stable circular polarization performance over the entire operating frequency band, laying a solid foundation for array beamforming. In this design, the element spacing of the subarray is set to 10.5 mm, which can effectively suppress the generation of grating lobes within the operating frequency band for the formation of a 16 × 16 element array.

The ceramic beamforming module is the core component for realizing multi-beam reception and electronic scanning functions. It integrates key functions such as front-end LNA, amplitude control, phase control and multi-beam synthesis. This module integrates the aforementioned multifunctional chips and power divider/combiner chips, and is fabricated via the HTCC process, thus achieving high interconnection density while featuring excellent thermal performance.

Based on the proposed architecture, each receiving subarray can independently generate eight beam signals and support a switchable DCP operating mode. By highly integrating radio frequency, control and power distribution functions within the ceramic module, the volume and interconnection complexity of the subarray-level system are significantly reduced, which facilitates modular expansion and further enhances the engineering feasibility of the entire array system.

### 3.3. Combining Board Design and Interconnection Scheme

**Figure 11:**
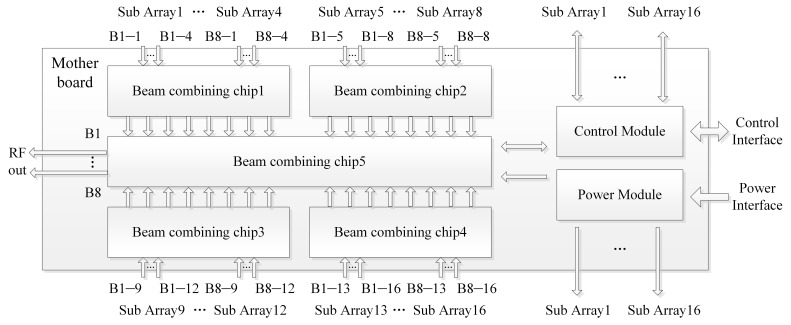
System schematic of the combining board.

**Figure 12:**
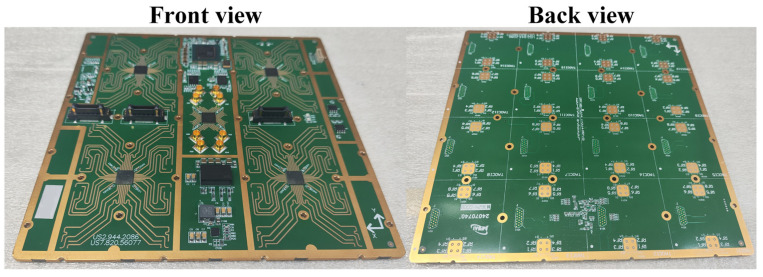
Photograph of the fabricated combining board.

Figure 11 and Figure 12 present the system principle block diagram and prototype photograph of the combining board, respectively. As the core control and aggregation unit of the proposed eight-beam DCP receiving phased array system, the combining board mainly performs signal combination, beam control, and system power supply functions. Its specific functions are summarized as follows. First, in terms of signal combination, the combining board implements eight groups of 16-to-1 RF signal combining, in which the signals corresponding to the same beam from the 16 receiving subarrays are further combined to form the eight output beams of the full array. Second, for beam control, the combining board is responsible for the decoding and distribution of beam control codes, thereby supporting the amplitude and phase control of each receiving subarray and enabling flexible beam reconfiguration of the array. In addition, the combining board provides a stable direct current (DC) power supply for all receiving subarrays to ensure the reliable operation of the entire system.

**Figure 13:**
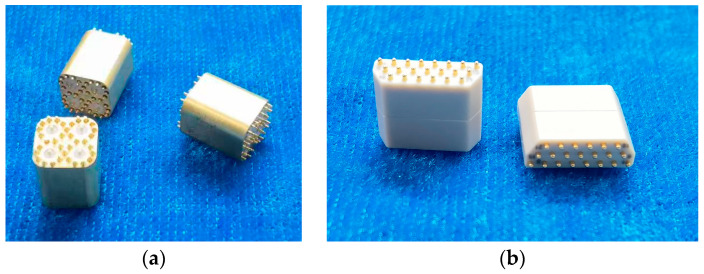
Vertical interconnection between the receiving subarrays and the combining board. (**a**) RF pogo pins; (**b**) Low-frequency pogo pins.

To further improve the integration level and reduce assembly complexity, traditional RF coaxial connectors are not adopted between the receiving subarrays and the combining board. Instead, as shown in Figure 13, RF pogo pins and low-frequency pogo pins are employed to realize vertical interconnection for RF signals, control signals, and power supply. This interconnection scheme effectively reduces the overall profile height while maintaining good RF performance and improving assembly consistency and practical feasibility.

## 4. Measurement and Results

**Figure 14:**
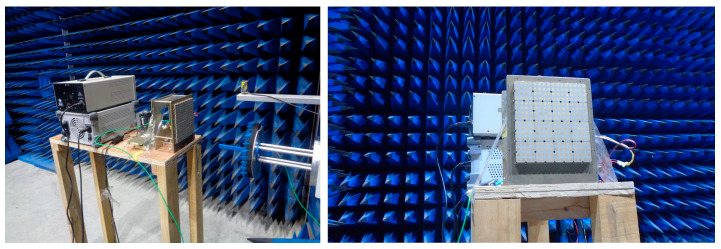
Measurement setup of the proposed array prototype in a microwave anechoic chamber.

To verify the design feasibility and engineering performance of the proposed low-cost 2D active receiving phased array system with reconfigurable dual-polarized eight beams, a 16 × 16 phased array prototype operating at 10.7–12.7 GHz was fabricated and implemented based on the HTCC process in this work. The overall size of the array is 168 mm × 168 mm. Both far-field and near-field tests of the phased array were conducted in a standard anechoic microwave chamber, and the test scene of the proposed prototype in the chamber is presented in Figure 14.

Prior to radiation pattern measurements, amplitude and phase calibration was performed to compensate for multi-channel mismatches. As illustrated in Figure 3, the array adopts an eight-beam independently controlled analog beamforming architecture, in which each beam is implemented using dedicated digitally controlled phase shifters and attenuators. The phase shifters have a step resolution of 5.625° and the attenuators provide a resolution of 0.5 dB. Calibration was conducted on a per-beam, per-element basis using near-field measurements, and amplitude as well as phase compensation coefficients were extracted for each antenna element and each polarization channel. The extracted coefficients were statically applied during the measurement process. Radiation patterns were then characterized separately for the eight reconfigurable dual-circularly polarized beams.

**Figure 15:**
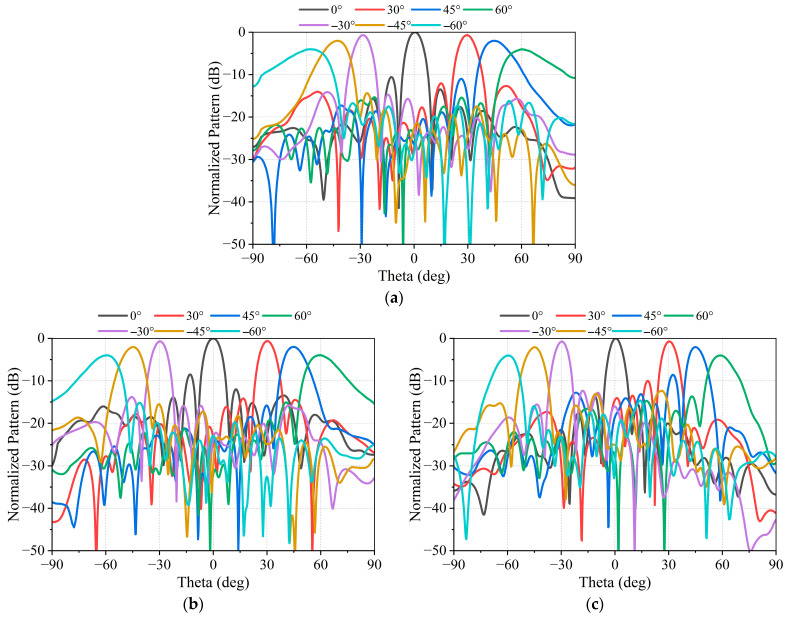
Measured scanning radiation patterns of the prototype operating in single-beam LHCP mode. (**a**) 10.7 GHz; (**b**) 11.7 GHz; (**c**) 12.7 GHz.

**Figure 16:**
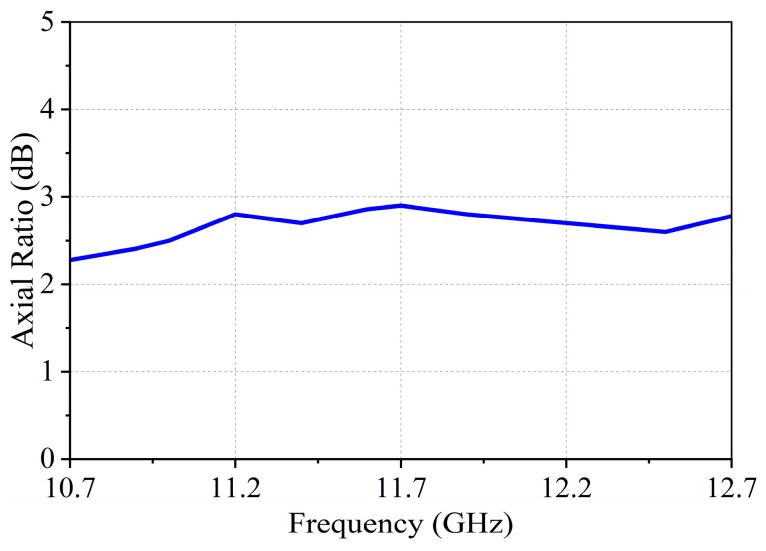
Measured axial ratio of the array.

Figure 15 presents the measured scanned radiation patterns of the array operating in the LHCP beam mode at three frequency points of 10.7 GHz, 11.7 GHz and 12.7 GHz. As shown in Figure 15, the array maintains stable beam scanning characteristics over the entire operating frequency band. When the scanning angle reaches 60°, the measured scanning gain degradation is less than 4 dB, indicating a good wide-angle scanning capability. Figure 16 presents the measured axial ratio of the array at boresight, demonstrating good circular polarization performance.

**Figure 17:**
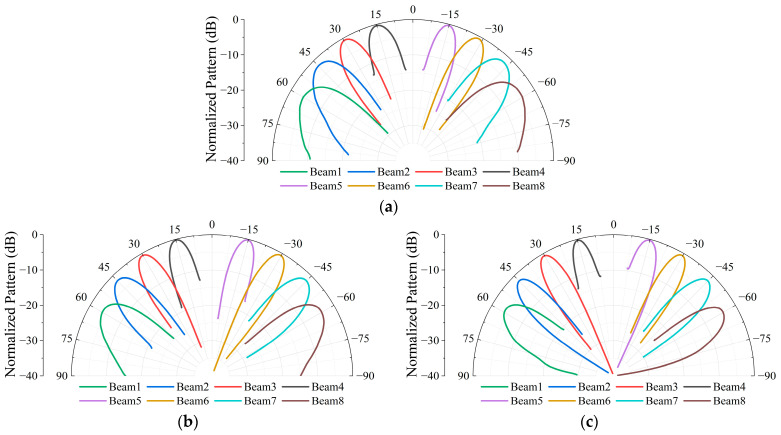
Measured radiation patterns with the array operating in eight-beam LHCP mode. (**a**) 10.7 GHz; (**b**) 11.7 GHz; (**c**) 12.7 GHz.

Figure 17 shows the measured radiation patterns of the array operating in the eight-beam LHCP mode simultaneously. It can be observed that the multiple beams exhibit good isolation and directional consistency, which verifies the effectiveness of the proposed beamforming architecture under the condition of simultaneous multi-beam operation.

To further evaluate the receiving performance of the proposed phased array system, a noise budget analysis of the RF front-end chain was conducted. The antenna radiation efficiency is approximately 0.9. The first-stage low-noise amplifier exhibits a noise figure of 1.3 dB with a gain of 20 dB, followed by an analog beamforming chip with a noise figure of 7.5 dB and a gain of 22 dB. The subsequent combining chip has a noise figure of 11.5 dB and provides a positive gain of 2 dB. According to Friis’ cascade noise formula, the overall system noise figure is primarily determined by the first two active stages. Since the cumulative gain of the LNA and beamforming chip exceeds 40 dB, the noise contribution from the subsequent combining stage and following RF components becomes negligible. Based on the above parameters, the calculated overall array noise figure is approximately 1.4 dB. Therefore, the receiving sensitivity of the proposed array is mainly governed by the front-end devices, and no significant noise degradation is introduced by the multi-beam combining architecture.

The G/T values of the proposed 16 × 16 phased array system at three frequency points were measured. The tests were conducted in an anechoic chamber with the antenna noise temperature *T*_ant_ set to 300 K. Table 1 summarizes the measured G/T values of the phased array system operating in the eight-beam mode. When the beam was scanned in the boresight direction, the measured maximum G/T value of Beam8 reached 10.9 dB/K at 10.7 GHz; the measured G/T values of both Beam2 and Beam8 hit 1.2 dB/K at 11.7 GHz; and the measured G/T values of both Beam2 and Beam7 also reached 1.2 dB/K at 12.7 GHz. The above results indicate that the proposed system can maintain stable receiving performance over the entire operating frequency band. As can be seen from Table 1, the G/T values remain favorable for the simultaneous eight-beam operation, which demonstrates that the proposed array architecture achieves multi-beam reception without introducing significant degradation in receiving sensitivity.

Table 2 compares the key performance metrics of several reported phased array receiving systems in recent years. It can be seen that Reference [20] proposed a reconfigurable eight-beam four-element phased array receiving system with a beam scanning range of only ±27°, and the G/T values of the system were not provided either. The 32 × 32-element dual-circularly polarized phased array system proposed in Reference [25] achieves a scanning angle of ±60°, yet only supports single-beam operation. In contrast, the phased array system proposed in this work enables simultaneous DCP eight-beam operation, with the beam scanning range reaching ±60° as well. Compared with the designs in References [20,25,26], the proposed general low-cost implementation framework for eight-beam DCP phased arrays based on the arbitrary analog beamforming network exhibits certain performance advantages and also demonstrates strong engineering practicability.

## 5. Conclusions

This work proposes a generic low-cost implementation framework for an eight-beam DCP array based on an arbitrary-form analog beamforming network. A Ku-band receiving phased array prototype was designed and fabricated to verify the feasibility and practicality of the proposed framework. Measurement results demonstrate that the prototype operates over the 10.7–12.7 GHz band and exhibits good beamforming performance. The array achieves a wide-angle scanning capability up to 60° with a scanning gain degradation of less than 4 dB. Moreover, the measured G/T values demonstrate that the proposed phased array framework incurs no significant degradation in receiving sensitivity when operating in the simultaneous eight-beam mode.

It should be noted that the proposed framework is not limited to receiving arrays and can also be extended to transmitting phased array systems. Future work will focus on the development and experimental validation of corresponding transmitting array prototypes based on the same framework.

## Figures and Tables

**Table 1:** The measured G/T values of the array under eight-beam operating conditions.

Beam Scan Angle	Beam Number	G/T@10.7GHz (dB/K)	G/T@11.7GHz (dB/K)	G/T@12.7GHz (dB/K)
boresight direction	Beam1	0.7	1.0	1.0
	Beam2	0.7	1.2	1.2
	Beam3	0.8	1.1	1.0
	Beam4	0.5	0.9	0.9
	Beam5	0.7	1.0	0.9
	Beam6	0.7	1.1	1.1
	Beam7	0.7	1.1	1.2
	Beam8	0.9	1.2	1.1

**Table 2:** Comparison of the proposed antenna array with some existing array.

	Frequency (GHz)	Number of Elements	Beam	Polarization	Scan Range (°)	Size (cm^2^)	G/T (dB/K, Measured)
[20]	7.5–9.0	2 × 2	8	Dual-Linear	±27	5.42 × 3.62	\
[25]	17.7–20.2	32 × 32	1	Dual-Circular	±60	24 × 28	6.22/6.51 (*T*_ant_ = 290 K)
[26]	29.5	8 × 8	4	Linear	±50	4.4 × 4.4	−7 (*T*_ant_ = 290 K)
Prop.	10.7–12.7	16 × 16	8	Dual-Circular	±60	16.8 × 16.8	0.9/1.2 (*T*_ant_ = 300 K)

## Data Availability

All data generated or analyzed during this study are included in this manuscript. There are no additional data or datasets beyond what is presented in the manuscript.
